# Photoinduced spin dynamics in a uniaxial intermetallic heterostructure $\hbox {TbCo}_2/\hbox {FeCo}$

**DOI:** 10.1038/s41598-020-72740-x

**Published:** 2020-09-25

**Authors:** Sergei Ovcharenko, Mikhail Gaponov, Alexey Klimov, Nicolas Tiercelin, Philippe Pernod, Elena Mishina, Alexandr Sigov, Vladimir Preobrazhensky

**Affiliations:** 1 MIREA - Russian Technological University, Moscow, Russia 119454; 2 Univ. Lille, CNRS, Centrale Lille, Yncréa ISEN, Univ. Polytechnique Hauts-de-France, UMR 8520 - IEMN, 59000 Lille, France; 3 Prokhorov General Physics Institute of RAS, Moscow, Russia 119991

**Keywords:** Optical spectroscopy, Magnetic properties and materials, Magnetic properties and materials, Phase transitions and critical phenomena

## Abstract

Intermetallic heterostructures of rare-earth and transition metals exhibit physical properties prospective for various applications. These structures combine giant magnetostriction, controllable magnetic anisotropy, magneto-optical activity and allow spin reorientation transitions (SRT) induced by magnetic field at room temperature. Here, we present the results of a study of spin dynamics induced by ultrafast optical excitation in the $\hbox {TbCo}_2\hbox {/FeCo}$ heterostructure. The time dependence of the light polarization rotation excited by a pump optical pulse with a duration of 35 fs was measured in the total range of the SRT created by external DC magnetic field. We found hysteretic dependence of the polarization rotation on magnetizing field that is specific for spin dynamics near SRT. Enhancement of the rotation is observed in the critical points of the SRT and near the points of magnetization switch from metastable to stable spin states. In the time-domain, two characteristic delays of 20 ps and 200 ps were found, corresponding to the maximum deviation of the light polarization after excitation. The first is explained by the precession motion of spins out of the plane of the structure. The latter is accounted for the spin in-plane deviation from its initial position and thermal relaxation of the anisotropy.

## Introduction

Strain control of magnetic states in magnetoelectric heterostructures is currently considered as a breakthrough in prospective nanoelectronic applications^1–4^. $\hbox {TbCo}_2/\hbox {FeCo}$ heterostructures have been proposed as basic component for straintronic magnetoelectric random access memory (MELRAM) with ultralow energy consumption^4–8^. The principle of operation of such devices is based on spin reorientation transitions (SRT) induced by short strain pulses generated by piezoelectric constituent elastically coupled with the structure. The switching rate between magnetic states in the MELRAM cells was estimated as fractions of a nanosecond. This rate, as well as the operating conditions of the combined magnetic/piezoelectric structure depend both on the rate of strain pulses generation in piezoelectric and on the dynamic properties of the spin system of the active magnetic layer. However, the experimental demonstration of magnetic switching in the magnetic $\hbox {TbCo}_2/\hbox {FeCo}$ layer has so far been performed only on a millisecond time scale. The sub-nanosecond spin dynamics was studied by ferromagnetic resonance method^9^ which applicability is limited to materials with a fairly small resonance line width. The most adequate method for studying spin dynamics is the magneto-optical pump-probe technique with the use of femtosecond laser pulses^10^. This technique was successfully applied for observation of ultrafast spin-reorientation transitions in materials with natural SRT induced by temperature variation^11–13^. An enhancement was reported of spin precession amplitude and intensity of THz emission excited by femtosecond pulse in the vicinity of SRT temperature. The conditions for this type of SRT are determined by the natural temperature range of the SRT in the materials, which is generally below or above room temperature, which severely limits applications.

Recently, a magnetooptical pump-probe technique has been used to study the dynamics in $\hbox {TbCo}_2/\hbox {FeCo}$ heterostructure with SRT induced by magnetic field^14^. However, in those studies, the anisotropy field $H_A$ of the sample was stronger than the available magnetizing field $H (H< H_A$). This prevented the achievement of optimal conditions for optical excitation of the spin system. The experimental results were interpreted using assumption of disruption of the uniaxial anisotropy by the femtosecond laser pulse. The similar assumption was proposed in Ref.^15^ for explanation of photo-induced excitations of the spin system in a magnetic dielectric.

In the present paper, we use the $\hbox {TbCo}_2/\hbox {FeCo}$ heterostructure with a weaker magnetic anisotropy than in Ref.^14,16^, which makes it possible to study the spin dynamics in the entire range of the SRT including the critical point $H=H_A$. The time-dependent change in the magneto-optical Kerr rotation of the polarization of the probe beam after the action of an ultrashort pump pulse is measured as a function of the magnetizing field.

The results of the measurements and calculations are compared; the latter were obtained using the Landau–Lifshitz–Gilbert (LLG) equation applied to a spin system excited by incoherent pulse disruption of the anisotropy field. We show that the proposed model describes adequately specific hysteretic dependences of photoinduced dynamic Kerr rotation observed experimentally and makes it possible to determine the characteristic time constants of the spin system under ultrafast optical impact. To our best knowledge, this is the first report on optical manipulation of the magnetization orientation in a uniaxial intermetallic heterostructure under SRT induced by magnetic field at room temperature.

## Results and discussion

**Figure 1:**
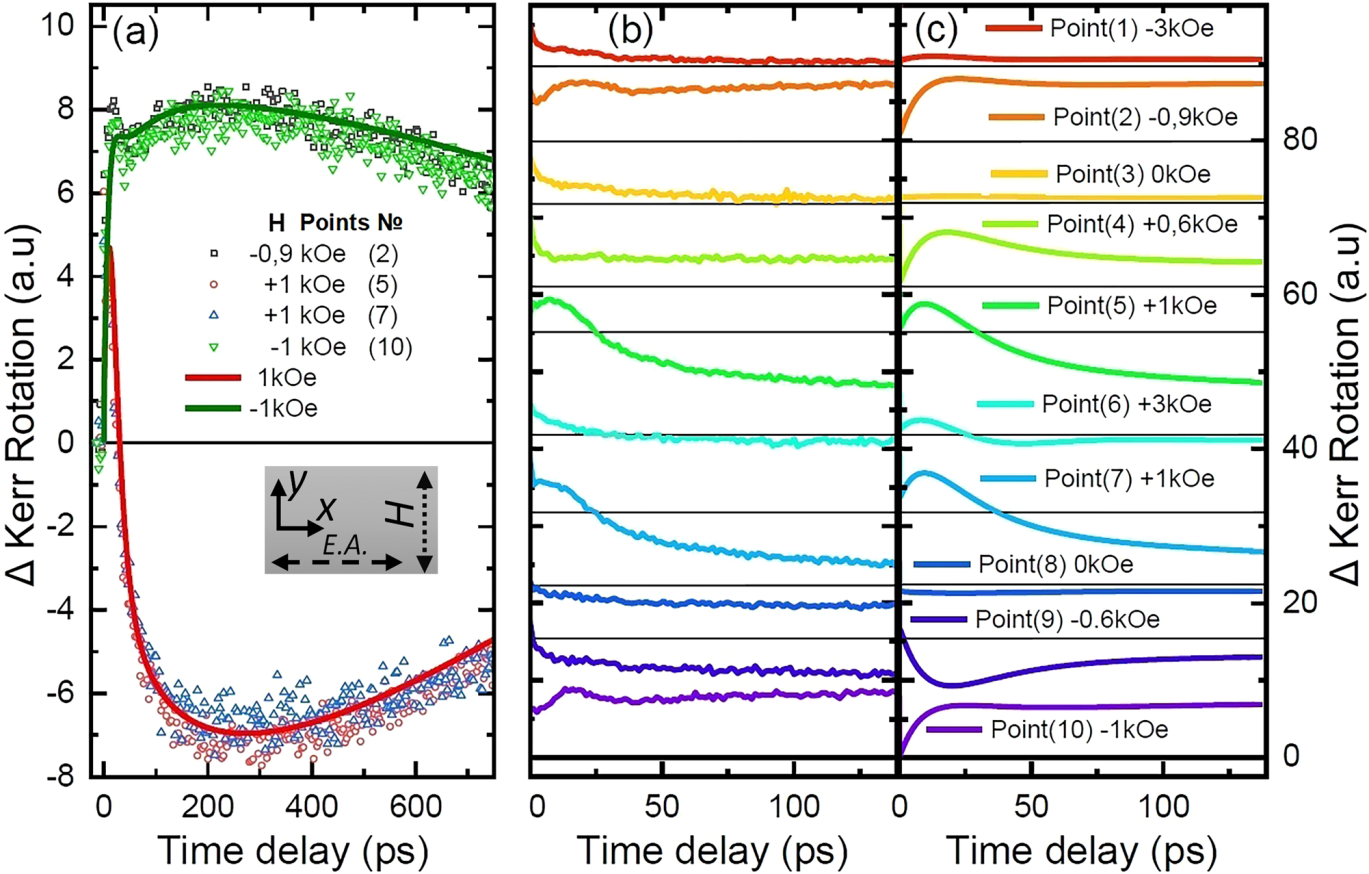
(**a**) Dynamics of the magneto-optical Kerr effect at the SRT points: experiment (points) and calculations (lines); Inset—mutual orientation of DC magnetic field *H*, Easy axis of magnetization (E.A.) regarding to the sample surface frame; (**b**) dynamics of the magneto-optical Kerr effect at different values of magnetic field; (**c**) calculation results. The horizontal lines show the zero level of the photoinduced signal for each curve. In the experiments, direction of magnetic field sweep was − 3 kOe $\rightarrow +3\hbox { kOe }\rightarrow -3\hbox { kOe}$. Each value of *H* is assigned the number of characteristic point, which appears in Fig. 2 as well.

**Figure 2:**
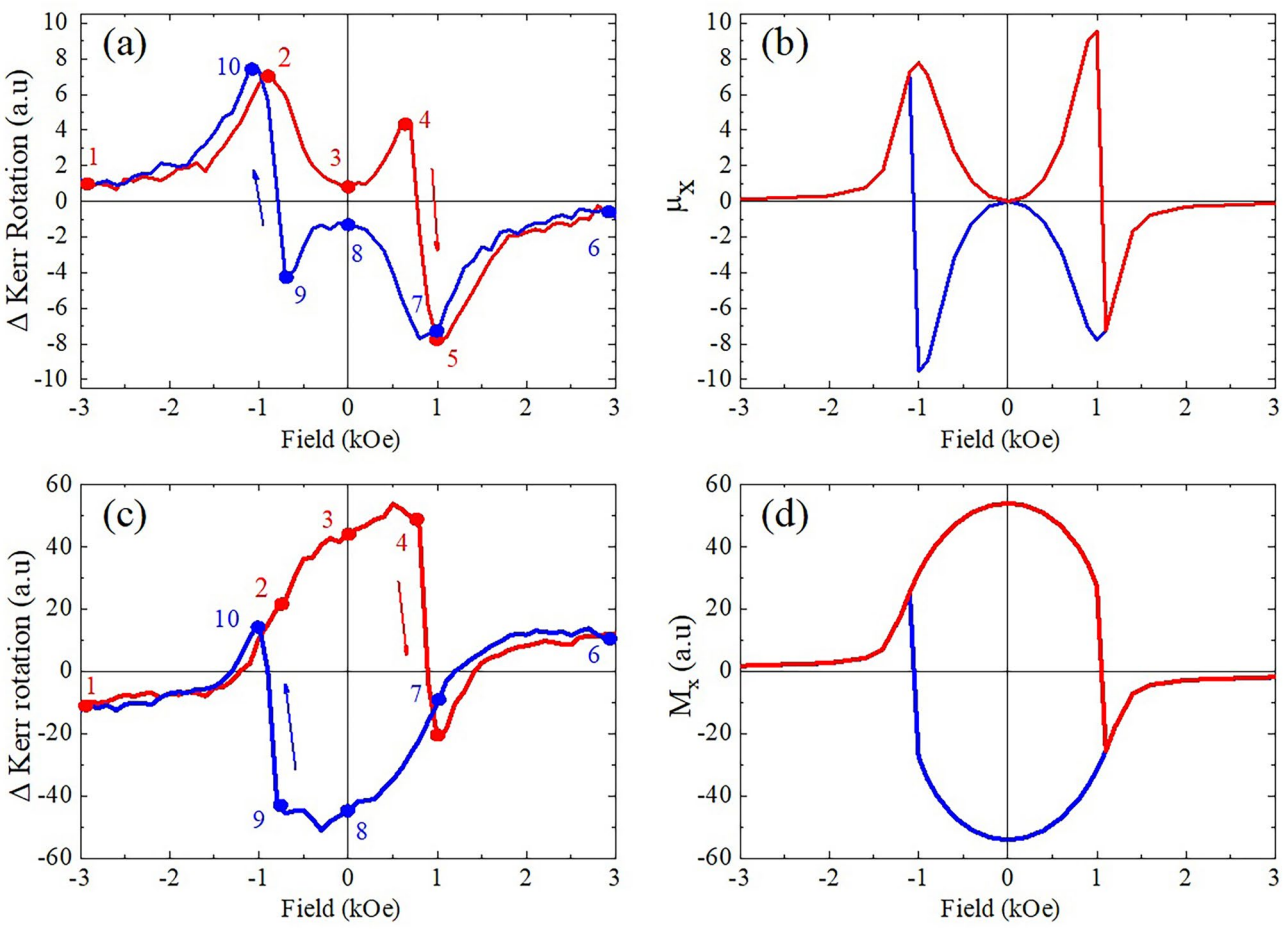
Dynamic hysteresis loops of the magneto-optical Kerr effect detected at the time delay $t_d = 150\hbox { ps}$ after pump pulse: (**a**) experiment, (**b**) calculation; static hysteresis loops of the magneto-optical Kerr effect without exposure to pumping radiation: (**c**) experiment, (**d**) calculation. Characteristic points of the loop are indicated with numbers which are used in Fig. 1 as well.

**Figure 3:**
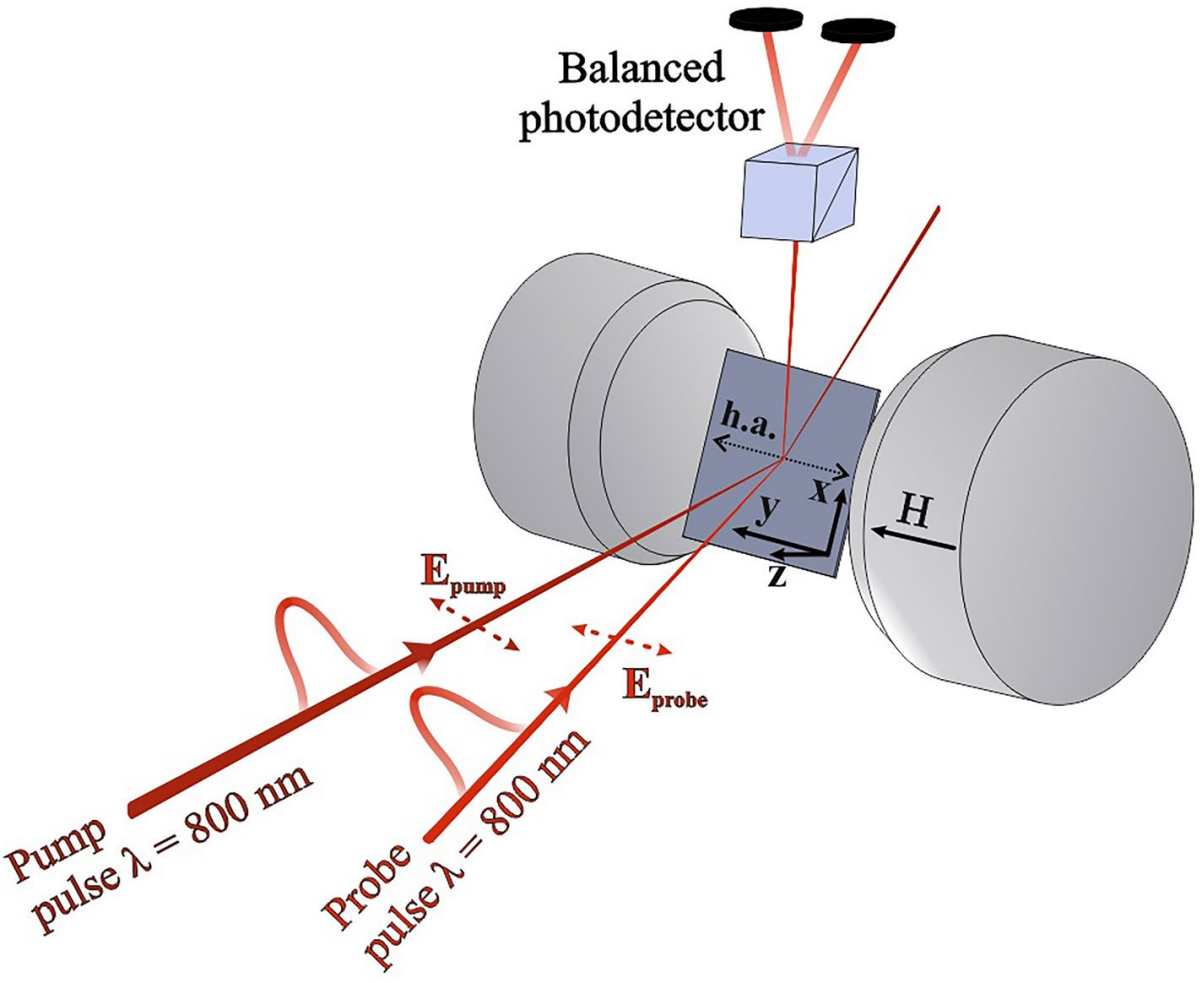
Experimental geometry: h.a. means hard magnetization axis.

The main results of observation of spin dynamics excited by ultrafast optical pump in the uniaxial heterostructure $6\times [\hbox {TbCo}_2$(2.4 nm)/FeCo(1 nm)] are shown in Figs. 1 and 2. Some details of the sample preparation technology are described in the “Methods” Section. For pump-probe experiments, the sample was placed in a DC magnetic field directed in plane of the structure normally to the easy axis of anisotropy (see inset in Fig. 1a). After impact of the pump pulse, the time dependent Kerr effect was detected with the probe beam incident at 45 degrees to the sample plane. The plane of incidence was parallel to the easy axis (see Fig. 3 in Method Section). Figure 1a shows deviation of the Kerr rotation angle from its static value in the entire time interval of observation $0< t_d < 750\hbox { ps}$. Two extrema can be distinguished in the figure. The first one appears at the delay time of the probe pulse $t_{d1} = 20\hbox { ps}$ relatively to the pump pulse, and the second one is displayed near $t_{d2} = 200\hbox { ps}$. Panel b in Fig. 1 demonstrates the details of the experimental Kerr rotation dynamics over the short time interval $0< t_d < 140\hbox { ps}$ for different values of the magnetizing field. From this panel it becomes clear that the extremum at $t_{d1}$ appears only for the selected values of magnetic field: at − 0.9 kOe, $+$ 1kOe, while sweeping magnetic field in forward direction, and at $+$ 1 kOe, − 1 kOe while sweeping in backward direction.

In order to identify the Kerr rotation behavior patterns while magnetic field has been sweeped, the dynamic hysteresis loops (HL) have been measured using stroboscopic technique at the fixed delay time. Figure 2a shows the dynamical HL measured at $\hbox {t}_d = 150\hbox { ps}$, which possesses several characteristic points. In particular, at points 2, 4, 5, 7, 9, 10, maximum deviations of the Kerr rotation were observed, indicating the most effective impact of the pump on the magnetic system of the sample at the corresponding values of the external magnetic field. In saturation (points 1 and 6), the value of dynamic Kerr rotation is much lower than in maxima. From points 4 to 5 and from 9 to 10 abrupt hops of magnetization are observed.

Due to the measurement technique, the observed dynamic HL shows the pump induced magnetization only. It lies on top of the background of unperturbed magnetization HL. The latter, which is the static one, can be measured independently with the pump beam completely blocked. The result is shown in Fig. 2c. Analogously to the dynamic loop, abrupt hops of magnetization are observed from points 4 to 5 and from 9 to 10. In contrast to dynamic HL, the maxima of magnetization are observed at zero magnetic field. In saturation (points 1 and 6), the value of Kerr rotation is much lower than in maxima, again similar to the dynamic HL.

To understand the experimental results and extract quantitative information, a numerical model was developed based on the LLG equation. The equation was presented in terms of angular variables $\varphi$ and $\theta$ determining projections of the magnetic moment $\hbox {M}_x=\hbox {M}\sin (\theta )\cos (\varphi )$, $\hbox {M}_y=\hbox {M}\sin (\theta )\sin (\varphi )$, $\hbox {M}_z=\hbox {M}\cos (\theta )$:

1 $$\begin{aligned}&\frac{\partial \phi }{\partial t}=\frac{\gamma M}{\Delta }\left[ \sin {\theta \frac{\partial F}{\partial \theta }}-{\alpha \frac{\partial F}{\partial \varphi }}\right] ,\nonumber \\&\frac{\partial \theta }{\partial t}=\frac{\gamma M}{\Delta }\left[ -\sin {\theta \frac{\partial F}{\partial \phi }}-{\alpha \sin ^2\theta \frac{\partial F}{\partial \theta }}\right] \end{aligned}$$

were *F* is the free energy density, $\gamma$ is gyromagnetic ratio, $\alpha$ is the spin relaxation parameter, $\Delta = {M^2}(1+\alpha ^2)\sin ^2\theta .$

In the framework of hypothesis of photoinduced disruption of magnetic anisotropy, we include in the free energy of the film the anisotropy energy with time dependent parameter $H_A(t)$ and energy of magnetic moment interactions with external and demagnetizing fields, determined by $H_x$, $H_y$, and $2{\pi }M^2$, respectively:

2 $$\begin{aligned} F=-\frac{1}{2}MH_A(t)\cdot \sin ^2\theta \cdot \cos ^2\theta -H_xM\cdot \sin \theta \cdot \cos \varphi -H_yM\cdot \sin \theta \cdot \sin \varphi +2\pi M^2\cdot \cos ^2\theta \end{aligned}$$

From the analysis of the experimental results, we introduce two relaxation parameters, $\eta _1$ and $\eta _2$:

3 $$\begin{aligned} H_A(t)=H_{A0}(1-\xi _1e^{\eta _1t}-\xi _2e^{\eta _2t}). \end{aligned}$$

They reflect thermalization of electronic system due to electron-phonon interaction and the thermal relaxation in the phonon system, respectively.

We assume that the *z* axis is perpendicular to the film plane, and the easy magnetization axis (EA) is parallel to the x axis of the film in accordance with the geometry shown in Fig. 3. The external field is applied parallel to the hard magnetization axis y (HA) with slight (about $1^{\circ }$) deviation to *x* axis in order to avoid uncertainty in the direction of magnetic moment rotation in the angular phase of SRT, in which magnetic moment forms an angle with a magnetizing field.

In the numerical solutions of the Eq. (1) we first find for $\xi _{1,2}=0$ the equilibrium in plane orientation, described by $\varphi _0$ as function of external field *H*. The values $M=800\hbox { emu/cm}^3$ and $H_{A0}= 1.2\hbox { kOe}$ were taken for calculations from the data of magnetization measurements by vibration sample magnetometer (VSM). The angle $\varphi _0$ was defined as the limit to which the solution of Eq. (1) tends for a sufficiently long time (about 2 ns). Figure 2d shows calculated projection $M_x=M\cos \varphi _0$ which is responsible for the static longitudinal Kerr effect. The obtained hysteresis loop corresponds to the results of magnetooptic experiment presented in Fig. 2c. The switching of the magnetic moment in fields $H_y = \pm \hbox { 1 kOe}$ reflects the transition from metastable to stable states formed due to small tilt of the magnetic field from a strict orientation along the hard axis.

The values $\varphi _0$ and $\theta _0=\frac{\pi }{2}$ are used as initial conditions for calculation of time dependent dynamic components of magnetic moment projections $\mu _x(t)=M_x(t)-M_x(0)$ and $\mu _z(t)=M_z(t)$. In order to determine contributions of each component to joint longitudinal and polar Kerr effect $\mu _{eff}(t)=\mu _x(t)+k\cdot \mu _z(t)$, the static rotations in magnetic field parallel to the plane and tilted out of plane were measured. The measurement data yielded $k=6$. The results of calculations of $\mu _{eff}(t)$ are presented in Figs. 1a,c and 2b,d. To obtain the time dependences consistent with the experimental observations, the following relaxation constants and parameters of the photoinduced decrease of the anisotropy field were used: $\alpha = 0.4$, $\eta _1=0.6\hbox { 1/ns}$, $\eta _2=0.1 1/\hbox {ps}$, $\xi _1=0.3$, $\xi _2=0.7$. The rapid variation of $\mu _{eff}(t)$ in the time interval $0<t_d< 150\hbox { ps}$ is mainly controlled by spin and anisotropy relaxation parameters $\alpha$ and $\eta _2$ as well as by disruption factor $\xi _2$.In the long time interval $300<t_d< 750\hbox { ps}$, the slow magnetic moment variation is mainly determined by the anisotropy relaxation time $\eta _1^{-1}$, which can be attributed to a thermal process.

Under the assumption of the anisotropy field disruption by the pump, the numerical simulation of the spin dynamics reveals the nature of the extremum at 20 ps as precession motion with exit of the magnetic moment from the plane of the structure. This motion is associated with polar Kerr effect. The results of calculations in the Fig. 1c are in satisfactiry agreement with the experimental data in the Fig. 1b. As an exception, the dynamic Kerr rotation accompanying uniform precession is absent in the experimental curves in Fig. 1b for specific switching points (4) and (9). Presumably, the reason for this lies in the appearance of inhomogeneous magnetic states, through which the magnetic moment switches from a metastable state to a stable one. Such “premature” switching before the attainment of absolute instability is characteristic of first-order phase transitions in magnets. The 200 ps extremum is caused by relatively slow in-plane rotation and relaxation of the magnetic moment. This movement generates a dynamic projection of magnetization onto the easy axis and contributes to the longitudinal Kerr effect.

Dynamic hysteresis loop in Fig. 2a,b strongly correlates with variation of static projection of magnetic moment on easy axis detected by longitudinal Kerr effect presented in Fig. 2c,d. When the strength of magnetic field applied normally to the easy axis exceeds the anisotropy field ($H>H_A$), magnetization is saturated along the field. When magnetic field decreases lower than $H_A$ , magnetic moment tilts towards the anisotropy axis. The point $H=H_A$ separates collinear and angular phases of SRT and corresponds to the critical point of the second order phase transition characterized by anomalous increase of sensitivity of the spin system to external perturbations. This feature is reflected in Fig. 2a in points 2 and 5.

The small tilt of the magnetic field to the easy axis makes unequal the energy of the two bistable states. The angular phase with the magnetic moment deviation in the same direction as the field tilt remains stable, while the phase with opposite direction of the moment deviation becomes metastable. Changing the field sign from positive to negative makes metastable the previously stable orientation. An increase in the negative magnetic field results in an abrupt switch from a metastable state to a stable one. The switch of the magnetic moment projection on the easy axis introduces asymmetry to the Kerr rotation angle at the points (7) $+$ 1kOe and (10) − 1kOe in Fig. 1a. The difference between the states (7) and (10) is reflected in the asymmetry of each brunch of the hysteresis loops in Fig. 2 a,c. The field strength at which the switch occurs is the second critical point of the SRT. Figure 2a,b demonstrates enhancement of the Kerr rotation effect due to the photo-induced spin excitation near the switching points (4, 5 and 9, 10) regarding to that at saturation.

In conclusion, we present the results of comprehensive experimental and theoretical study of spin system response to ultrafast optical impact in the in-plane-uniaxial intermetallic heterostructure $\hbox {TbCo}_2/\hbox {FeCo}$. The presence of two features simultaneously, each of two individually may be inherent in other materials, makes the studied effects and the studied heterostructures quite unique. Firstly, all observations relate to room temperature and secondly, the external magnetic field is the driving force of the SRT.

The results obtained by the optical pump probe technique demonstrate strong enhancement of the dynamic Kerr-rotation of the probe polarization at the values of the magnetizing field critical for SRT. Two kinds of critical points are fixed. In the first one, enhancement is typical for the critical point of the second order phase transitions, in which the order parameter, i.e. the angle of the spin deviation in our case, varies continuously. In the second one, the enhancement occurs near abrupt switch of magnetization from metastable to stable state.

The maxima of Kerr rotation are found at characteristic time delays between the probe and pump pulses equal to 20ps and 200ps. Comparison with the results of numerical simulations within the LLG theory (Fig. 1c) allows us to associate these characteristic delays with the precession deviation of the magnetic moment outside the plane of the structure and with relatively slow rotation in the plane, respectively.

We propose a mechanism of interaction of an optical pulse and a spin system, which consists in the thermal disruption of the anisotropy field. We prove that this effect alone fully explains the observed experimental results. Comparison of the experimental data with the calculations also revealed the values of spin relaxation parameter and thermal relaxation characteristics of anisotropy. The latter may be used as a guide, in particular, when constructing prospective heterostructures for straintronic MELRAM.

## Methods

### The sample fabrication technology

The multilayer film $6\times$[TbCo2(2.4 nm)/FeCo(1 nm)] was deposited on a silicon substrate by RF sputtering technology. The sputtering was carried out under static magnetic field that allows for fabrication of heterostructure with controllable magnetic anisotropy^17^. Here the sample with uniaxial anisotropy of effective field about 1.2 kOe was fabricated for studies of spin dynamics in the entire range of SRT angular phases using available magnetic field strength of a few kOe. Saturation magnetization and the anisotropy field were measured by VSM ADE EV 9.

### Optical experiment

The pump-probe scheme of the setup for ultrafast excitation and detection of spin dynamics using magnetooptic Kerr-effect is presented in Fig. 3.

The technique is similar to that used in Ref.^15^. The regenerative amplifier (RA) was pumped by Ti:Sa femtosecond laser TiF-20F (Avesta project, Russia) provided a 35 fs laser pulse with a repetition rate of 3 kHz at a central wavelength of 800 nm. Beams were focused onto the spot with $\hbox {D}_{pump} = 35\hbox { mkm}$ and $\hbox {D}_{probe} = 20\hbox { mkm}$. Pump density was $7\hbox { mJ/cm}^2$, probe density 10 times smaller. All measurements were carried out in reflection geometry. Linear p-polarization was chosen for both pump and probe pulses. The plane of incidence was normal to the sample surface and parallel to the easy magnetization axis that allowed observation of meridional and polar Kerr-effect when magnetic moment deviates in- and out of the sample plane. The angle of incidence of the probe beam was equal to 45 degrees. The sample was settled between the cores of the electromagnet providing a DC magnetic field up to 6.5 kOe in the plane of the sample. To register the deviation of magnetooptical Kerr rotation caused by optical pumping, a balanced photodetector based on a Wollaston prism with two photodiodes rotating around the optical axis and recording the orthogonal polarization components was used. The pump beam formed a small angle with the normal and was rejected from detector. The signal was obtained by synchronous detection using a Lock-in amplifier SR830. Variations of magnetic field strength and time delay between pump and probe pulses allowed for observation of time dependent reaction of the spin system on laser pump illumination and hysteretic dependence of Kerr-rotation during the total cycle of the SRT.
